# Population-based survival analysis of primary spinal chordoma in the US from 2000 to 2020

**DOI:** 10.1007/s11060-024-04807-y

**Published:** 2024-09-27

**Authors:** Kevin E. Agner, Michael C. Larkins

**Affiliations:** 1 The Ohio State University College of Medicine, 370 W. 9th Avenue, Columbus, OH 43210 USA; 2 East Carolina University Brody School of Medicine, 600 Moye Blvd, Greenville, NC 27834 USA; 3 Department of Emergency Medicine, Boonshoft School of Medicine at Wright State University, 2555 University Blvd, Fairborn, OH USA

**Keywords:** Spinal chordoma, Surgical oncology, SEER program, Population-level analysis, Histologic cancer analysis

## Abstract

**Purpose:**

Chordomas are rare malignant tumors that occur primarily in the axial skeleton. We seek to analyze trends affecting five-year overall survival (5y OS) among patients with primary spinal chordomas (PSC) of the vertebrae and sacrum/pelvis.

**Methods:**

The Surveillance, Epidemiology, and End Results (SEER) Program was used to identify patients with PSC (ICD-O-3 histology codes 9370/3, 9371/3, and 9372/3) of the spine or sacrum/pelvis. Multivariate and univariate survival analyses were conducted to assess demographic, disease, or treatment characteristic trends.

**Results:**

Eight-hundred-ninety-six patients diagnosed with PSC were identified. Patients 0–54 years at diagnosis had improved 5y OS compared to those either 55–69 years (HR = 1.78; *p* = 0.046) or those between 70 and 85 + years (HR = 3.92; *p* < 0.001). Histology impacted 5y OS: Cox regression demonstrated variance among the three histologies assessed (*p* < 0.001), while univariate analysis demonstrated patients with dedifferentiated chordoma (1.0% of cohort; 33.3% [1.9,64.7]) and chondroid chordoma (2.0% of cohort; 52.5% [26.1,78.9]) had decreased 5y OS compared to those with general chordoma (72.2% [68.8,75.6]; *p* < 0.001). Nonmarried patients had decreased 5y OS on univariate analysis (65.2% [59.4,71.0] versus 76.2% [72.0,80.4]), with widowed patients being the primary driver of this on subanalysis. Treatment with gross total resection was associated with increased 5y OS (HR = 0.22, *p* < 0.001), as was treatment with radiotherapy (HR = 0.69, *p* = 0.030).

**Conclusion:**

Patient age and marital status were significant demographic factors associated with changes in 5y OS among those with PSC. PSC histology is a potentially important prognostic factor in the management of disease.

## Introduction

Chordomas are rare, slow-growing, low-grade malignant tumors originating from remnants of the embryonic notochord [1, 2]. These tumors predominantly occur along the axial skeleton, most frequently in the sacral region (55%), followed by the skull base (35%), and vertebral column (10%; 1,2). Although generally slow growing, chordomas can be locally invasive and aggressive, infiltrating proximal muscles, nerves, and vascular structures. The annual incidence rate of new chordoma diagnoses is approximately one to two cases per million each year, with a slightly higher frequency in males than females [2].

Previous SEER database research has identified that age, race, tumor size, extent of resection, and tumor stage are important prognostic factors for primary spinal chordomas. Older patients tend to have poorer survival outcomes due to increased comorbidities and compromised physiological conditions [1–7]. Additionally, a larger tumor diameter is also associated with a poorer prognosis [5]. A variety of studies have demonstrated the association of surgical resection with improved survival, with those who undergo gross total resection (GTR) showing better outcomes than those who undergo subtotal resection (STR) or do not receive surgery at all [3, 5–7]. A 2018 study using the SEER database found that surgical resection for sacral tumors was significantly associated with improved survival [2].

Despite the growing body of research on chordomas, there is still much to learn about the impact of various factors on patient survival. Further investigation is needed to better understand the influence of demographic characteristics, treatment modalities, and other prognostic indicators, on patient outcomes. To date, there have been no multi-center studies of primary spinal chordoma that explore tumor histology or the impact of median household income on overall survival.

This study aims to explore this gap by conducting a retrospective epidemiologic survival study of primary spinal chordomas. Building upon previous research using the SEER database, this study will analyze the specific characteristics of primary spinal chordomas and their associations with patient demographics, treatment modalities, histological subtype, primary location, and survival outcomes.

## Materials and methods

### Patient identification

This retrospective study utilized data queried from the National Cancer Institute’s Surveillance, Epidemiology, and End Results (SEER) 17-registry Incidence database, which lists cases diagnosed between the years 2000 and 2020 [8]. Patients diagnosed with primary spinal chordoma (PSC) were identified using the International Classification of Diseases for Oncology, Third Edition (ICD-O-3) histology codes 9370/3: Chordoma, NOS, 9371/3: Chondroid chordoma, and 9372/3: Dedifferentiated chordoma, with the further specification of the primary site as the spine (C41.2) or sacrum/pelvis (C41.4 and C76.3) through the appropriate SEER site recode. Additionally, all cases were required to have a behavior code ICD-O-3 characterized as “Malignant.” The variables of interest were selected based on findings from previous studies and treatment factors associated with the standard of care [1, 5]. These variables are also the standard variables available via the SEER Program.

### Data analysis

Data was analyzed in SPSS (Version 29.0; Armonk, NY: IBM Corp.). A p-value of < 0.05 was used as the cutoff for statistical significance; 95% confidence intervals reported in brackets [95% CI]. Multivariate analysis was conducted via Cox regression based on five-year overall survival (5y OS). Kaplan-Meier (KM) survival curves were generated and compared using log-rank analysis for univariate and subanalysis. Surgical codes were determined based on the 2022 SEER Staging Manual [9]. Incomplete/missing entries were omitted from each individual analysis conducted.

## Results

### Cohort overview

Eight-hundred-ninety-six patients were identified with Primary Spinal Chordoma (PSC). Median five-year age bracket was 60–64 years, while cumulative five-year overall survival was 71.4% [68.0%,74.8%]. A summary of patient demographic and disease characteristic information can be found in Table 1. Patient age was divided into three approximately equal groups to allow for strong statistical comparison.

**Table 1:** Demographic, disease, and treatment information for patients diagnosed with primary spinal chordoma

Variable	Number (% of Cohort; *n* = 896)
*Age at Diagnosis*	
0–54 Years	287 (32.0%)
55–69 Years	302 (33.7%)
70–85+	307 (34.3%)
*Sex*	
Female	359 (40.1%)
Male	537 (59.9%)
*Race*	
White	765 (85.4%)
Asian or Pacific Islander	87 (9.7%)
American Indian/Alaska Native	2 (0.2%)
Black	32 (3.6%)
Unknown	10 (1.1%)
*Primary Site*	
Spine	383 (42.7%)
Sacrum/Pelvis	513 (57.3%)
*Histology*	
Chordoma, NOS	869 (97.0%)
Chondroid Chordoma	18 (2.0%)
Dedifferentiated Chordoma	9 (1.0%)
*Marital Status*	
Single	334 (37.3%)
Married	516 (57.6%)
Unknown	46 (5.1%)
*Median Household Income*	
< $40,000-$79,999	426 (47.5%)
$80,000-$120,000+	470 (52.5%)
*Stage*	
Localized	368 (41.1%)
Regional	303 (33.8%)
Distant	75 (8.4%)
Unknown	150 (16.7%)
*Radiotherapy (RT)*	
No RT	434 (48.4%)
RT	434 (48.4%)
Unknown	28 (3.1%)
*RT-Surgery Sequence*	
Before Surgery	35 (3.9%)
After Surgery	247 (27.6%)
Before and After	24 (2.7%)
N/A	590 (65.8%)
*Chemotherapy*	
No Chemotherapy	864 (94.4%)
Chemotherapy	50 (5.6%)
*Surgical Procedure*	
None	237 (26.5%)
Subtotal Resection	315 (35.2%)
Gross Total Resection	308 (34.4%)
Unknown	36 (4.0%)

*NOS: Not Otherwise Specified

Patient demographic, disease characteristic, and treatment information for patients diagnosed with Primary Spinal Chordoma in the US between 2000 and 2020, as identified via the Surveillance, Epidemiology, and End Results (SEER) Program

### Multivariate analysis

Cox regression analysis with respect to five-year overall survival was conducted for patients identified with PSC and can be found in Table 2. Increasing age was associated with decreased survival, with patient age between 70 and 85 + years at diagnosis associated with significantly decreased 5y OS (hazard ratio (HR) = 3.92, *p* < 0.001).Both chondroid chordoma (HR = 3.56, *p* = 0.015) and dedifferentiated chordoma (HR = 5.16, *p* < 0.001) histologies were associated with decreased 5y OS compared to those patients with unspecified histology. Treatment with radiotherapy was associated with increased 5y OS as well (HR = 0.69, *p* = 0.030). Surgical procedures notably improved survival, with gross total resection (GTR) showing the greatest benefit (HR = 0.22, *p* < 0.001). Additionally, advanced disease stage was associated with increased mortality, as patients with distant disease had a higher hazard of death (HR = 2.87, *p* < 0.001). The primary site variable showed significance with spinal tumors having decreased survival compared to the pelvic/sacral tumors (HR = 1.48, *p* = 0.036).

**Table 2:** Cox regression analysis of five-year overall survival among patients with primary spinal chordoma

Variable	*p*-value	Hazard Ratio
*Age at Diagnosis*	< 0.001	N/A
55–69 vs. 0–54 and 70–85+	0.046	1.78
70–85 + vs. 0–54 and 55–69	< 0.001	3.92
*Sex*	0.35	1.18
*Race*	0.97	N/A
Asian or Pacific Islander vs. White and Black	0.81	0.94
Black vs. White and Asian or Pacific Islander	0.93	1.05
*Median Household Income ($80*,*000-$120*,*000 + vs. < $40*,*000-$79*,*999)*	0.74	1.06
*Marital Status (Married vs. Single)*	0.14	0.77
*Histology*	< 0.001	N/A
Chondroid Chordoma vs. NOS and Dedifferentiated Chordoma	0.015	3.56
Dedifferentiated Chordoma vs. NOS and Chondroid Chordoma	< 0.001	5.16
*Radiotherapy Yes vs. No*	0.030	0.69
*Chemotherapy Yes vs. No*	0.72	1.12
*Surgical Procedure*	< 0.001	N/A
STR vs. None and GTR	< 0.001	0.48
GTR vs. None and STR	< 0.001	0.22
*Stage*	< 0.001	N/A
Regional vs. Localized and Distant	0.008	1.67
Distant vs. Localized and Regional	< 0.001	2.87
*Primary Site (Pelvis/Spine)*	0.036	1.48

Results from Cox regression analysis for five-year overall survival. Significant variables include age at diagnosis, median household income, histology, surgical procedure performed, stage, and primary site. Comparisons within categories are listed in the rows below italicized variables if relevant. Comparisons among three or more categories within a variable do not have an associated hazard ratio

### Univariate survival analysis

**Fig. 1:**
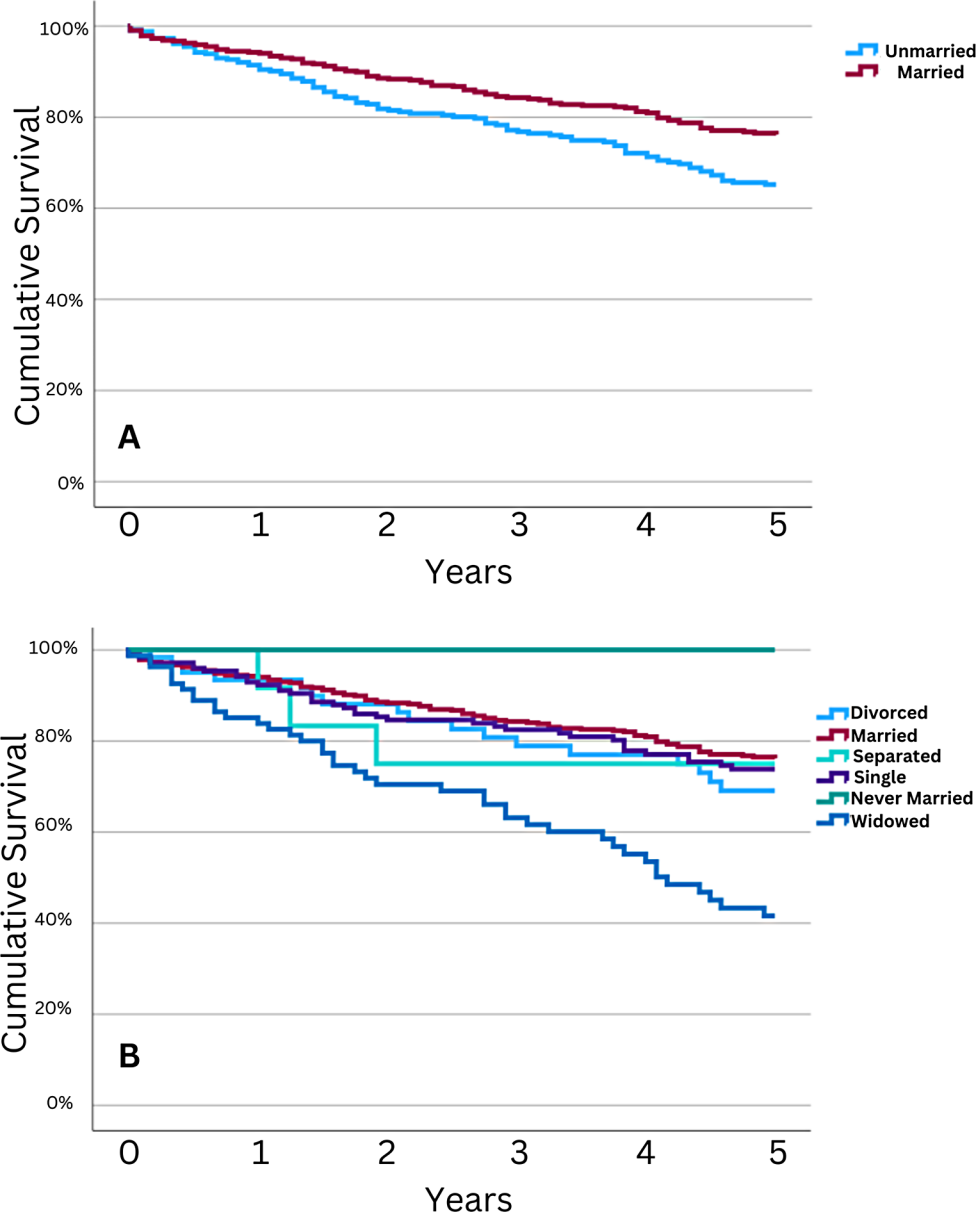
Five-year OS in Primary Spinal Chordoma Patients Stratified by Marital Status. Kaplan-Meier curves depicting OS for patients diagnosed with primary spinal chordoma stratified by marital status at diagnosis. **A**: Comparison of unmarried patients to those that were married at diagnosis. Unmarried patients had decreased 5y OS (65.2% [59.4,71.0]) compared to those that were married at diagnosis (76.2% [72.0,80.4]). **B**: Comparison of patients further stratified by marital status (separated, single (never married), etc.). Widowed patients had significantly decreased 5y OS (41.6% [29.4,53.8]) compared to all other categories, and comparison amongst these groups was statistically significant (*p* < 0.001)

Univariate comparison of five-year overall survival (5y OS) can be found in Table 3. This analysis showed that age at diagnosis significantly impacted five-year OS, with patients aged 0–54 years and 55–69 years at diagnosis seeing increased 5y OS compared to those aged 70–85+ )84.5% and 79.0% versus 51.2%, respectively; *p* < 0.001). Concerning disease histology, patients with either chondroid chordomas or dedifferentiated chordomas had decreased 5y OS compared to those with unspecified histology (52.5% and 33.3% versus 72.2%, respectively; *p* < 0.001). Decreasing 5y OS was seen with increasing severity of summary stage, with those with local disease faring the best at 78.2%. Non-married patients had decreased 5y OS (65.2%) compared to married patients (76.2%; *p* = 0.001; see Fig. 1A). Subanalysis of marital status demonstrated widowed patients had significantly decreased 5y OS (41.6%) compared with all other marital statuses; married patients had the highest 5y OS at 76.2% (see Fig. 1B).

**Table 3:** Univariate five-year overall survival (OS) analysis among patients with primary spinal chordoma

Variable	OS [95% CI]	Five-year OS log-rank *p*-value
*Age at Diagnosis*		< 0.001
0–54 Years	84.5% [79.7,89.3]	
55–69 Years	79.0% [73.8,84.2]	
70–85+	51.2% [44.8,57.6]	
*Sex*		0.53
Female	70.1% [64.7,75.5]	
Male	72.3% [68.1,76.5]	
*Race*		0.41
White	70.9% [67.3,74.5]	
Asian or Pacific Islander	70.9% [59.7,82.1]	
American Indian/Alaska Native	N/A*	
Black	83.1% [67.3,98.9]	
*Histology*		< 0.001
Chordoma NOS	72.2% [68.8,75.6]	
Chondroid Chordoma	52.5% [26.1,78.9]	
Dedifferentiated Chordoma	33.3% [1.9,64.7]	
*Marital Status*		0.001
Non-married	65.2% [59.4,71.0]	
Married	76.2% [72.0,80.4]	
*Income*		0.081
< $40,000-$79,999	68.6% [63.8,73.4]	
$80,000-$120,000	74.4% [69.8,79.0]	
*Stage*		< 0.001
Localized	78.2% [73.2,83.2]	
Regional	73.4% [67.8,79.0]	
Distant	41.4% [27.2,55.6]	
*Chemotherapy*		0.016
No Chemotherapy	72.3% [68.9,75.7]	
Chemotherapy	55.3% [39.1,71.5]	
*Surgical Procedure*		< 0.001
None	48.6% [41.0,56.2]	
Subtotal Resection	73.6% [68.0%,79.2]	
Gross Total Resection	85.6% [81.2,90.0]	
*Primary Site*		0.79
Sacrum/Pelvis	71.0% [66.6,75.4]	
Vertebrae	72.0% [66.8,77.2]	
*Radiotherapy (RT)*		0.33
No RT	70.6% [65.8,75.4]	
RT	71.8% [67.0,76.6]	
*RT Order*		< 0.001
RT before surg	93.0% [83.4,100.0]	
RT after surg	81.1% [75.5,86.7]	
RT before + after surg	88.4% [72.8,100.0]	
N/A	65.4% [61.0,69.8]	
*Marital Status Subanalysis*		< 0.001
Divorced	69.1% [56.5,81.7]	
Married	76.2% [72.0,80.4]	
Separated	75.0% [50.0,100.0]	
Single (never married)	73.8% [66.4,81.2]	
Unmarried	N/A*	
Widowed	41.6% [29.4,53.8]	

*One case identified

This table summarizes factors affecting five-year survival in spinal chordoma patients. Age, stage, histology, chemotherapy, and surgical procedure are significantly associated with survival. Overall log-rank p-value is presented in the rightmost column

Treatment with chemotherapy was associated with decreased 5y OS (55.3% versus 72.3%). Comparison between surgical techniques (none versus subtotal resection versus gross total resection, GTR) demonstrated GTR to offer superior 5y OS (85.6%; *p* < 0.001). Analysis of patient sex, race, median household income, disease location, and treatment with radiotherapy (RT) did not demonstrated differences in 5y OS. While treatment with RT did not demonstrate survival benefit, when assessed, neoadjuvant RT offered the highest benefit to 5y OS (93.0%), while patients not treated with both surgery and RT fared the worst (65.4% 5y OS).

### Demographic factor analysis

The majority of patients diagnosed with primary spinal chordoma were between 55 and 85 + years old, with a noticeable decrease in survival rates as age increased. Multivariate Cox regression analysis found a statistically significant association between increasing age and decreased survival, with hazard ratios (HR) of 1.78 for the 55-69-year-old group and 3.92 for the 70–85 + year-old group, compared to the 0-54-year-old group (*p* < 0.001; see Table 2). Kaplan-Meier analysis showed a significant difference in five-year survival rates between the age groups, with the oldest age group (70–85+) having the poorest survival (51.2% [44.8,57.6], *p* < 0.001; see Table 3). No difference in 5y OS was present with respect to sex (*p* = 0.35), race (*p* = 0.97), median household income (*p* = 0.74), or marital status on multivariate analysis (*p* = 0.14; see Table 2). On univariate analysis, marital status (married versus non-married) was significant (*p* < 0.001), with married patients having increased 5y OS (76.2% versus 65.2%, respectively). Further analysis demonstrated widowed patients to be a significant driver of this trend, with 45y OS of 41.6% reported for this group.

### Histology and stage

**Fig. 2:**
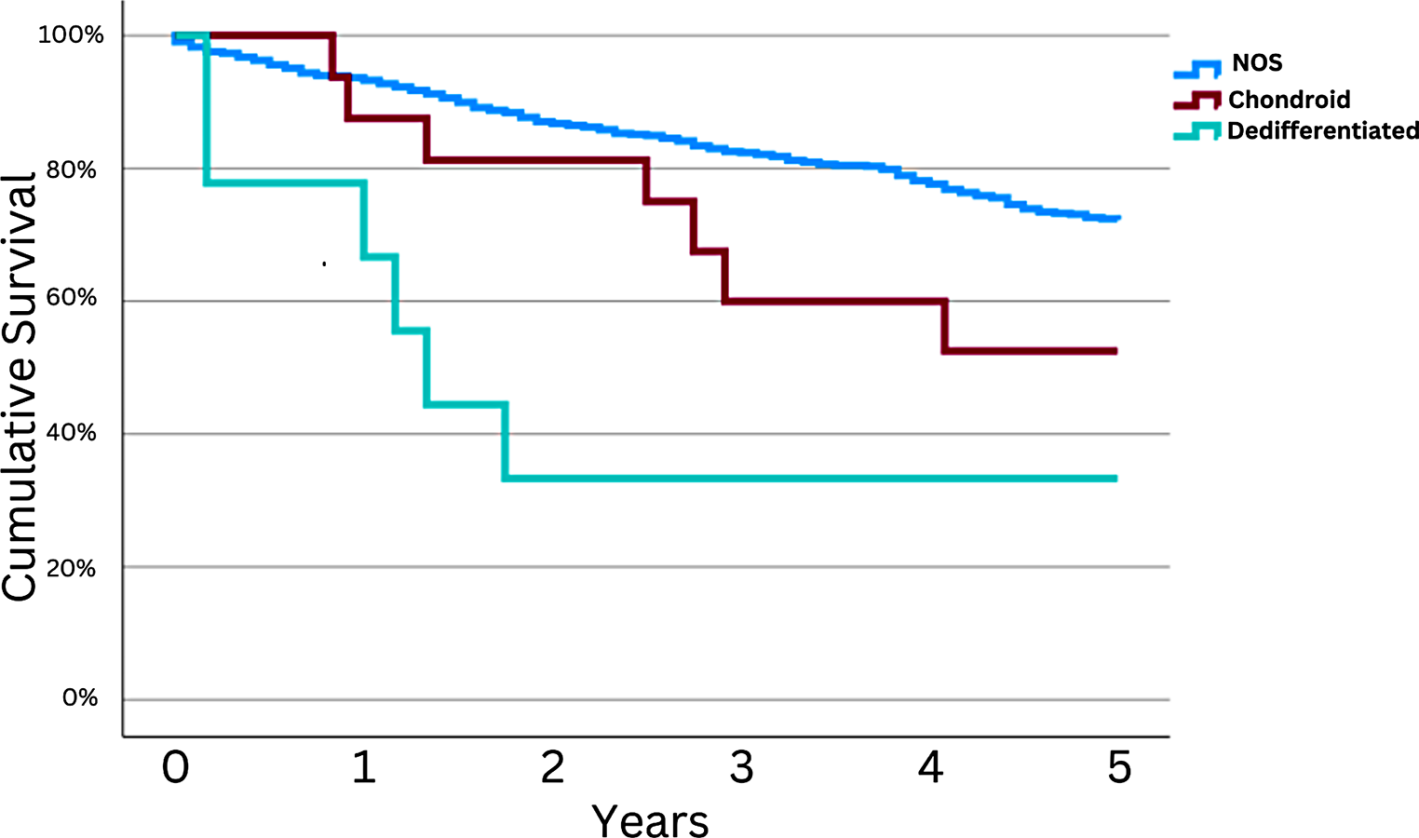
Univariate Survival OS Curve for Primary Spinal Chordomas Stratified by Histological Type. Kaplan-Meier OS curve depicting overall survival for Primary Spinal Chordoma patients stratified by histological types: Not Otherwise Specified, Chondroid, and Dedifferentiated Chordoma, demonstrating a significant difference in survival outcomes across time points (*p* < 0.001). 5y OS for Chordoma, NOS: 72.2% [68.8,75.6]; Chondroid Chordoma: 52.5% [26.1,78.9]; Dedifferentiated Chordoma: 33.3% [1.9,64.7]

The vast majority of patients (97.0%) were diagnosed with Chordoma NOS with a smaller number of patients having Chondroid Chordoma (2.0%) or Dedifferentiated Chordoma (1.0%). Cox regression analysis showed overall histological significance with five-year OS (*p* < 0.001), with both chondroid (HR = 3.56, *p* = 0.015) and dedifferentiated chordoma (HR = 5.16, *p* < 0.001) linked to higher mortality compared to unspecified chordoma histologies (see Table 2). This trend was further assessed on univariate analysis, with patients diagnosed with dedifferentiated chordomas having the lowest associated 5y OS (33.3%) compared to 52.5% for chondroid chordomas and 72.2% for unspecified chordoma histologies (*p* < 0.001; see Fig. 2).

The majority of patients in the cohort were diagnosed with localized disease (41.1%), followed by regional (33.8%) and distant (8.4%) disease. Cox regression analysis showed a statistically significant association between stage and survival (*p* < 0.001, see Table 2). This analysis found that distant disease, compared to local disease, was associated with increased mortality (HR = 2.87; *p* < 0.001). Patients with regional disease also had decreased 5y OS comparatively (HR = 1.67, *p* = 0.008). This trend was evident on univariate analysis as well, with patients with distant disease having significantly decreased 5y OS (41.4%) compared to both those with local and regional disease.

### Radiotherapy (RT) and chemotherapy

Almost half of the patients (48.4%) received RT for treatment. This was associated with increased 5y OS (HR = 0.69, *p* = 0.030), though on univariate analysis this trend did not persist (*p* = 0.33).

Only a small percentage of patients (5.6%) received chemotherapy. Cox regression analysis did not find a statistically significant association between chemotherapy use and overall survival (*p* = 0.72, see Table 2). On univariate analysis, treatment with chemotherapy was associated with decreased 5y OS, with those treated with chemotherapy having a 5y OS of 55.3% versus 72.3% for those not receiving chemotherapy (*p* = 0.016).

### Surgical procedure analysis

The study found that a majority of patients underwent either subtotal resection (STR) or gross total resection (GTR), with 35.2% and 34.4% receiving those respective treatments. Both STR and GTR had significantly reduced hazard ratios compared to those who received no surgery, with hazard ratios of 0.48 (*p* < 0.001) and 0.22 (*p* < 0.001), respectively (see Table 2). Univariate analysis demonstrated the same trend, with those treated with GTR faring better than those treated with STR (5y OS = 85.6% versus 73.6%, respectively).

### Primary site

Analysis of PTC primary site demonstrated the majority of tumors were located in the sacrum/pelvis (57.3%) compared to the spine (42.7%; see Table 1). Cox regression analysis found a significant association between primary site and survival (*p* = 0.036), with a higher hazard ratio associated with patients diagnosed with a tumor in the pelvis compared to those with vertebral tumors (HR = 1.48, see Table 2). However, this trend did not persist on univariate survival analysis (*p* = 0.79).

## Discussion

This analysis demonstrated increased five-year overall survival among patients with PSC on Cox regression analysis with respect to increasing patient age, disease histology, increasingly severe disease stage, PSC of the sacrum/pelvis compared to PSC of the vertebrae, and lack of surgical treatment. On multivariate analysis, increased patient age was associated with increased mortality (*p* < 0.001), with patients in the youngest age group (0–54 years of age at diagnosis) faring the best compared to both patients aged 55–69 years (HR = 1.78; *p* = 0.046) and those aged 70–85+ (HR = 3.92; *p* < 0.001). On univariate analysis, patients aged 70–85 + at diagnosis (5y OS: 51.2% [44.8,57.6]) had significantly increased mortality as compared to those aged 55–69 years (79.0% [73.8,84.2]) and those aged 0–54 years (84.5% [79.7,89.3]; *p* < 0.001). This trend is consistent with previous studies [1–3, 5–7], and is likely secondary to increased frailty and morbidities associated with patients diagnosed with cancer at an older age. These findings emphasize the importance of early diagnosis and intervention for chordoma, particularly in older patients, as they are at an increased risk for poorer survival.

While marital status was not significant on multivariate analysis (*p* = 0.14), on univariate analysis among all marital status subtypes (divorced, married, single (never married), etc.) demonstrated variation in 5y OS, with widowed individuals demonstrating significantly reduced 5y OS (41.6%) compared to those either separated, married, or single. Married patients had the highest survival (76.2% [72.0,80.4]. This finding suggests that socioeconomic factors, beyond the impact of age, may influence survival in chordoma patients.

The study found that a diagnosis of dedifferentiated chordoma was significantly associated with decreased survival (HR = 5.16; *p* < 0.001). This aligns with previous research highlighting the aggressive nature of this histological subtype in 18 subjects [10]. The five-year survival rate for dedifferentiated chordoma were 33.3% [1.9,64.7]. Chondroid chordomas were also associated with decreased 5y OS when compared to unspecified chordoma histologies (52.5% [26.1,78.9]. These findings underscore the importance of accurate histopathological classification for guiding treatment strategies and prognostication. This information could potentially be incorporated into the treatment and prognosis of patients with PSC and potentially chordomas in general.

The study also confirmed the critical role of surgical intervention in improving survival outcomes. Patients who underwent either subtotal resection (STR) or gross total resection (GTR) showed significantly reduced hazard ratios compared to those who received no surgery (*p* < 0.001). The hazard ratios were 0.48 (*p* < 0.001) for STR and 0.22 (*p* < 0.001) for GTR. Furthermore, Kaplan-Meier analysis demonstrated a significant difference in five-year survival rates based on surgical procedure (*p* < 0.001), with the highest 5-year survival rate observed in patients who underwent GTR (85.6% [81.2,90.0]), followed by STR (73.6% [68.0,79.2]), and the lowest in those who received no surgery (48.6% [41.0,56.2]). These results are consistent with previous studies emphasizing the importance of surgical resection in improving survival for spinal chordoma [3, 5–7]. Radiotherapy (RT) was also associated with increased survival (HR = 0.69, *p* = 0.030), and while this trend did not persist with univariate analysis (*p* = 0.33), subanalysis of patients treated with both surgery and RT demonstrated treatment with both modalities was associated with increased 5y OS, with neoadjuvant RT having the highest 5y OS (93.0% [83.4,100.0]). This is contrary to previous literature, which has not established a clear association between treatment with both RT and surgery to hold mortality benefit [11, 12].

The study also found a significant association between stage and overall survival (*p* < 0.001), highlighting the importance of early detection and intervention, a sentiment echoed by previous research emphasizing the importance of early detection and intervention for chordoma [1–3]. Patients diagnosed with distant disease had decreased survival both on multivariate (HR = 2.87, *p* < 0.001) and univariate (5y OS = 41.4% [27.2,55.6], *p* < 0.001) compared to those with localized disease. While chemotherapy use was statistically insignificant in the Cox regression model (*p* = 0.72, Table 3), Kaplan-Meier analysis showed a significant difference in 5-year survival between those who received chemotherapy fairing worse (55.3% [39.1,71.5]) than those who did not (72.3% [68.9,75.7], *p* = 0.016). There is an anticipated negative selection bias for patients receiving only chemotherapy as treatment; furthermore this phenomenon is possibly due to its usage among a small percentage of patients who likely had advanced disease, a finding also noted in previous studies [13].

Finally, the study found that a tumor located in the spine was associated with a significantly higher hazard ratio compared to tumors located in the sacrum/pelvis region (HR = 1.48, *p* = 0.036). This finding is consistent with a previous study that found a similar association between tumor location and survival [5]. This may be secondary to the complex anatomy of the sacrum, potentially greater risk of local invasion, and the difficulty in achieving complete resection in this region.

While this study offers valuable insights, it is important to acknowledge its limitations. The study relied on a multi-center database, potentially limiting the depth of information about individual patient-level factors. The national survival data does not include information related to hospital quality or patient volume for this condition. However, it is important to note that better outcomes for rare cancers are typically achieved in tertiary institutions with high patient volumes and specialized expertise [14, 15]. The ability to assess for differences between centers is not possible secondary to the de-identification of patient data within the SEER Program. Additionally, data on treatment-associated morbidity and patient frailty are lacking, although the authors recognize the significant influence of these factors on the choice of treatment modalities. Furthermore, the SEER Program does not provide specific details on the doses or agents used in the treatments, particularly with RT and chemotherapy. Finally, the curative intention of all treatments is assumed, as the SEER Program does not give any information on the curative or palliative intent with any therapies.

## Data Availability

The datasets generated during and/or analysed during the current study are available in the SEER Program repository, https://seer.cancer.gov/.
